# Non–ECG-gated cardiac CT angiography in acute stroke is feasible and detects sources of embolism

**DOI:** 10.1177/17474930231193335

**Published:** 2023-08-22

**Authors:** Peter Lee, Gurmohan Dhillon, Marina Pourafkari, Dominique DaBreo, Zardasht Jaff, Ramana Appireddy, Albert Jin, Lysa Boissé Lomax, Bryce A Durafourt, John Gordon Boyd, Amir Reza Nasirzadeh, Donatella Tampieri, Shirin Jalini

**Affiliations:** 1 School of Medicine, Queen’s University, Kingston, ON, Canada; 2 Department of Radiology, Kingston Health Sciences Centre, Queen’s University, Kingston, ON, Canada; 3 Division of Cardiology, Department of Medicine, Kingston Health Sciences Centre, Queen’s University, Kingston, ON, Canada; 4 Division of Neurology, Department of Medicine, Kingston Health Sciences Centre, Queen’s University, Kingston, ON, Canada

**Keywords:** Cardiac CT, imaging, hyperacute stroke, cardio-aortic embolism, ischemic stroke

## Abstract

**Background:**

A significant portion of cryptogenic stroke is hypothesized to be secondary to cardiac embolism. However, transthoracic echocardiogram is usually delayed after stroke, and more detailed cardiac imaging is not routinely done.

**Aims:**

This study aimed to determine whether non–ECG-gated cardiac CT angiography (cCTA) during hyperacute stroke would provide diagnostic quality images and act as an adjunct modality of cardiac imaging to detect sources of emboli.

**Methods:**

In this single-center prospective cohort study, modified Code Stroke imaging was implemented with a 64-slice CT scanner, where the longitudinal axis of CT angiography was extended from the carina to the diaphragm. The primary outcomes of image quality, recruitment feasibility, impact on hyperacute time metrics, and additional radiation dose were assessed. Secondary outcomes consisted of detection of high-risk cardiac sources of embolism, mediastinal or lung pathology, and impact on etiologic classification.

**Results:**

One hundred and twenty eligible patients were enrolled, of which 105 (87.5%) had good/moderate quality images for motion artifact and 119 (99.2%) for contrast opacification. Total CT time, door-to-needle time, and door-to-groin puncture time were unchanged with the addition of cCTA. Eighty-nine patients received a final diagnosis of ischemic stroke, of which 12/89 (13.5%) had high-risk cardioembolic findings on cCTA. Incidental findings, such as pulmonary embolism (PE) (7/89, 7.9%) and malignancy (6/89, 6.7%), were observed. cCTA led to changes in management for 19/120 (15.8%) of all patients, and reclassification of stroke etiology for 8/89 (9%) of patients.

**Conclusions:**

Non–ECG-gated cCTA can be feasibly incorporated into Code Stroke and provide diagnostic quality images without delays in hyperacute time metrics. It can detect high-risk cardiac sources, and other findings impacting patient care. This may help reclassify a subset of cryptogenic stroke cases and improve secondary prevention.

## Introduction

The cause of approximately one-third of ischemic strokes remains unknown despite routine investigations.^1^ Imaging and clinical characteristics of these strokes often suggest a distant embolic source, leading to the entity called embolic stroke of undetermined source (ESUS).^2^ Indiscriminate anticoagulation in this patient population has not proved superior for secondary stroke prevention as per recent clinical trials,^3,4^ with recurrence rates of 4–5% per year with either antiplatelet or anticoagulation therapy.^5^

Stroke etiology in the ESUS population is likely heterogeneous. It is postulated that only a portion of ESUS arises from cardiac embolism,^6^ through mechanisms such as paroxysmal atrial fibrillation (AF) or atrial cardiopathy. These are important to identify for three principal reasons. First, cardioembolic strokes are more severe and have higher mortality than other subtypes.^7^ Second, they have a relatively higher early recurrence rate.^7^ Third, anticoagulation is an effective strategy for secondary prevention.^8^ Therefore, it is important to refine investigations to expose the ESUS subpopulation with a potential cardiac substrate as early as possible.

CT angiography (CTA) of large vessels is recommended by all international guidelines in the setting of suspected stroke to guide hyperacute treatment.^9–11^ This sometimes unmasks stroke etiology, such as carotid stenosis, allowing for expedited implementation of targeted prevention.

In contrast, cardioembolic investigations, such as a transthoracic echocardiogram (TTE), are often delayed by days or weeks after index stroke.^12^ By this time, cardiac thrombi may no longer be detectable, especially if thrombolytics were administered.^13^ TTE also has limitations such as variable image quality,^14,15^ dependence on operator expertise,^15^ and inability to clearly visualize the left atrium (LA) and left atrium appendage (LAA), a common site of thrombus formation in AF, and atrial cardiopathy.^6,14^ Transesophageal echocardiogram (TEE), which offers gold standard visualization of the LA/LAA, is invasive, not widely available due to resource constraints,^14,15^ and may exacerbate dysphagia in stroke patients.^16^

Cardiac CT angiography (cCTA) is a potential alternative imaging modality to assess cardiac structures and address some of the limitations of echocardiogram. ECG-gated cCTA has been investigated for the detection of LA/LAA thrombus, showing comparable sensitivity and specificity to TEE, but may be challenging to practically implement in the hyperacute setting.^12,15^ A recent study performed a dedicated ECG-gated cCTA immediately after hyperacute stroke and showed a median increased scan time of 6 min.^12^ In contrast, non–ECG-gated cCTA can be more seamlessly incorporated without significantly increased imaging time or radiation dose and without additional injection of contrast. Recent data have suggested potential utility of non–ECG-gated cCTA in providing diagnostic quality images for detecting high-risk cardioembolic sources,^17–19^ but needs further investigation and replication.

## Aims and hypothesis

The primary objectives of this study are to assess the quality, feasibility, and safety of non–ECG-gated cCTA during Code Stroke imaging. The secondary objective is to assess its potential clinical utility. We performed a single-center prospective observational cohort study of obtaining diagnostic quality images of the heart by extending the *z*-axis coverage of non–ECG-gated cCTA performed during the imaging assessment of hyperacute stroke. We hypothesized this would offer diagnostic quality images and detect high-risk cardiac sources of embolism.

## Methods

### Study design

This was a single-arm descriptive cohort study of participants undergoing non–ECG-gated cCTA as part of neurovascular imaging at a Canadian comprehensive stroke center. The cohort was defined as participants presenting a Code Stroke. The exposure of interest was the non–ECG-gated cCTA. For the purpose of this study, patients presenting with acute stroke symptoms underwent a modified standard-of-care Code Stroke imaging protocol where the CTA field began at the diaphragm instead of the carina (see Supplemental Table 1 for CT protocol). Average heart rates were not formally captured and no heart-rate-lowering medications were administered. Images were obtained under free breathing using a GE Healthcare 64-slice Revolution HD 2016 CT scanner. Following image acquisition, multiplanar reconstructed images were obtained, and cCTA images were interpreted by fellowship-trained cardiothoracic radiologists.

### Setting

Kingston Health Sciences Center is a university teaching hospital affiliated with Queen’s University in Kingston, Ontario, Canada and a comprehensive stroke center providing care to ~500,000 population. Participants were prospectively enrolled over 10 months (March 2021 to December 2021) during regular working hours due to the requirement for timely reporting of the cCTA. Participants were followed up for up to six weeks post-symptom onset. Study outcomes were determined by cardiothoracic radiologists and stroke neurologists and data was collected by a research assistant in a case record form.

### Participants

Inclusion criteria were: (1) Age ⩾ 18 years, and (2) presenting as a Code Stroke to the emergency department during daytime hours. Exclusion criteria consisted of (1) contrast allergy, (2) evidence of intracerebral hemorrhage on CT head, and (3) strong clinical suspicion of stroke mimics during emergency evaluation. Eligible participants were enrolled to undergo cCTA as part of the modified Code Stroke imaging.

### Variables

Exposure of interest was the “non–ECG-gated cCTA” done as part of standard-of-care Code Stroke imaging. Outcomes are defined below.

#### Primary outcomes (imaging quality and recruitment feasibility)

These included: (1) Image quality of non–ECG-gated cCTA (see Figure 2(a)) as measured by *motion artifact* (based on confidence to exclude LAA/LA thrombus and assess LA morphology), and *contrast opacification* (based on LA and tubular ascending aorta). (2) Lack of delay in hyperacute stroke imaging time metrics (total CT time) and treatment metrics (door-to-needle and door-to-groin puncture times). (3) Recruitment feasibility outcomes: Number of cases that met inclusion and exclusion criteria and were recruited.

#### Secondary outcomes (clinical utility of non–ECG-gated cardiac CTA)

These included: (1) Rates of detecting high-risk cardiac sources of embolism, (2) Rates of detecting non-cardiac pathologies such as pulmonary or mediastinal pathology. (3) Impact on TOAST classification and patient management at discharge.

Cardiac thrombus was defined as a lobulated/ovoid low-density filling defect with < 100 hounsfield units (HU). Indeterminant/possible slow flow was defined as incomplete filling of the LAA and > 100 HU.

#### Primary safety outcomes

This consisted of an additional radiation dose from cCTA.

### Data sources and measurement

Imaging quality was assessed using a Likert-type scale (Table 1) by three fellowship-trained cardiothoracic radiologists (G.D., M.P., and D.B.). Kappa score was obtained based on two readers (G.D. and M.P.) blindly reviewing cCTAs for image quality and cardiac source of emboli. Safety data was collected from the radiation dosage report generated by the scanner. Data on clinical utility outcomes were collected from electronic health records, including imaging, cardiac testing, and consultation records.

**Table 1.** Assessment of image quality of non–ECG-gated cardiac CTA using a Likert-type scale.

Motion artifact quality assessment	
3: Good	Little to no blurring motion artifact resulting in high degree of confidence to exclude LAA thrombus.
2: Moderate	Fair degree of confidence to assess LAA despite some motion artifact. Good visualization with an echocardiogram is recommended to confirm.
1: Poor	Motion artifact precludes the ability to adequately assess LAA to comment on potential thrombus.
Contrast opacification	
3: Good	Hounsfield units (HU) > 250
2: Moderate	HU 150–250
1: Poor	HU < 150

CTA: CT angiography; LAA: left atrium appendage; ECG: electrocardiogram; HU: hounsfield units.

### Study size

As this was a study of a novel imaging paradigm with the goal to assess the quality, feasibility, and safety of cCTA during standard-of-care delivery, no sample size calculation was done.

### Standard protocol approvals, registrations, and patient consent

Ethics approval was obtained from the Queen’s University Health Sciences and Affiliated Teaching Hospitals Research Ethics Board (HSREB; #6030510), and a deferred consent process was implemented. Consent was obtained at the first available opportunity from the patient or substitute decision-maker.

### Statistical methods

Data were analyzed using descriptive statistics summarizing the distribution of findings relating to primary and secondary objectives. Microsoft Excel (Version 16.61.1, 2019) was used to calculate frequencies, percentages, means, medians, and interquartile ranges. Interrater reliability of image quality and cardiac source of emboli was assessed using the kappa score.

## Results

**Figure 1.**
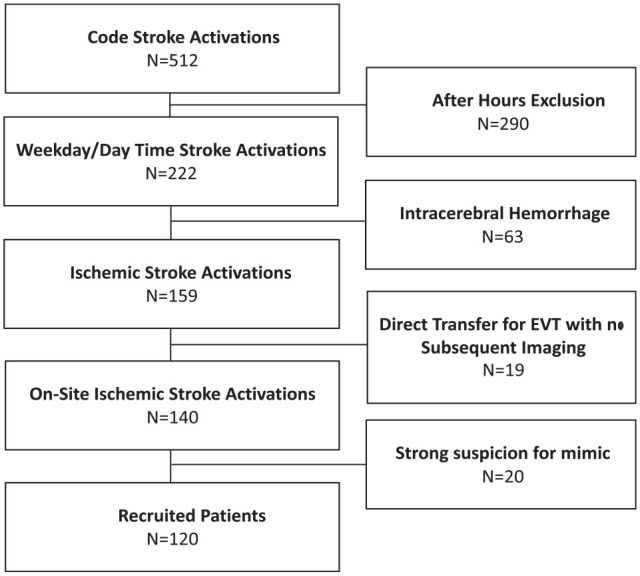
Patient recruitment for cCTA protocol. Over a 10-month period, 120 of 512 patients who presented as Code Stroke met the criteria and were recruited to undergo cCTA. Reasons for omission included (1) after-hours presentation, whereby radiological expertise was not present (57%), (2) intracerebral hemorrhage (12%), (3) imaging already completed (4%), and (4) strong suspicion of stroke mimics (4%).

Between March and December 2021, there were 512 Code Stroke activations. Of these, 120 patients were prospectively enrolled. Enrollment details are included in Figure 1.

### Demographic profile

Table 2 shows the baseline demographics of recruited patients. Of the 120 patients who underwent cCTA, 89 (74.2%) received a final diagnosis of ischemic stroke.

**Table 2.** Baseline demographics of recruited patients.

Characteristic	
Age, years, median (interquartile range)	73 (63–81)
Sex, male *n*, (%)	66 (55)
Diagnosis, *n*, (%)	89 (74.2)
Ischemic stroke	
Anterior circulation	79 (65.8)
Posterior circulation	9 (7.5)
Large vessel occlusion	1 (0.8)
Stroke mimic	31 (25.8)
Risk factors, *n* (%)	
Hypertension	89 (74.2)
Dyslipidemia	70 (58.3)
Smoking	107 (89.2)
Atrial fibrillation	32 (26.7)
Coronary artery disease	32 (26.7)
Diabetes	35 (29.2)
Baseline mRS, median (interquartile range)	1 (0–2)
Initial NIHSS score, median (interquartile range)	
All patients	5 (3–13)
Stroke patients	7 (3–15)
Hyperacute treatment	
Intravenous thrombolysis	20 (16.7)
Mechanical thrombectomy	22 (18.3)

NIHSS: National institutes of health stroke scale.

### Primary outcomes

#### Imaging quality of non-gated cardiac CTA

The primary outcome was interpretable image quality, as evaluated by cardiothoracic radiologists. Interrater reliability kappa score was 0.82 and 1.00 based on two readers (G.D. and M.P.) blindly reviewing cCTAs for image quality and cardiac source of emboli, respectively.

**Figure 2.**
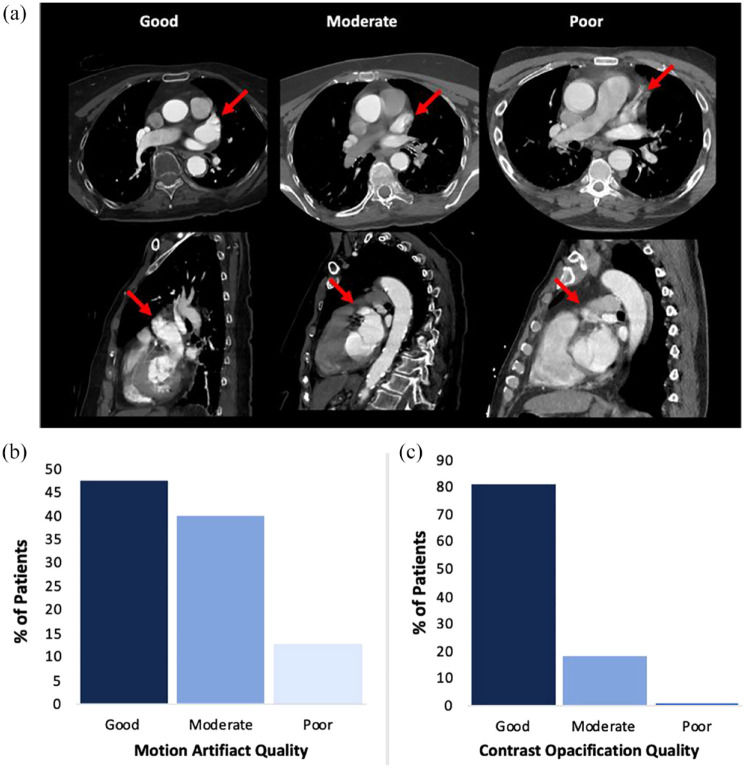
The imaging quality of non–ECG-gated cCTA in code stroke includes: (a) Examples of non–ECG-gated cCTA images graded based on motion artifact (confidence to exclude LAA thrombus and assess LAA morphology). Note: Arrow indicates LAA. (b) Motion artifact scores measured the degree that patient factors impacted image clarity. Good motion artifact scores were determined for 47.5% of patients (57/120), moderate for 40% (48/120), and poor for 12.5% (15/120) of patients. (c) Contrast opacification provided an indication of tissue border definition on CT. Good contrast opacification was observed for 80.8% of patients (97/120), moderate for 18.3% (22/120), and poor for 0.8% (1/120).

Image quality based on motion artifact was rated as good (57/120, 47.5%), moderate (48/120, 40%), or poor (15/120, 12.5%; Figure 2(b)). Contrast opacification was good in the majority of patients (97/120; 80.9%), moderate in 22/120 (18.3%), and poor in 1/120 (0.8%; Figure 2(c)).

#### Impact on time metrics

**Figure 3.**
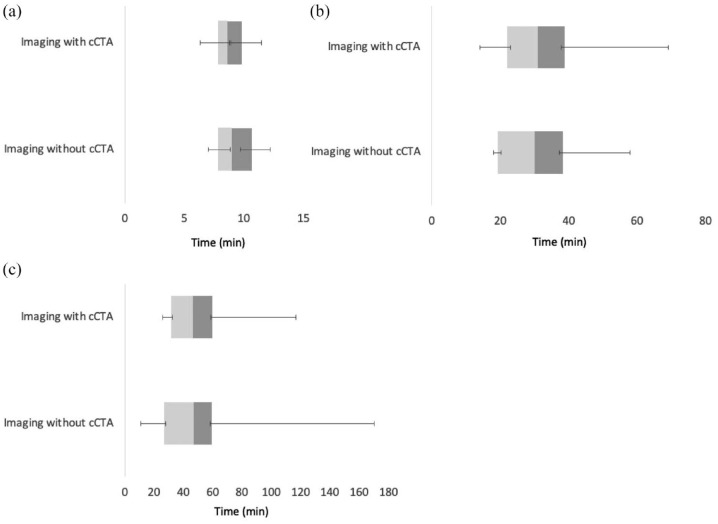
Impact of cCTA on time metrics of stroke imaging and treatment. (a) *Total CT time*. CT time, defined as the time between the first and last image, was unchanged when comparing patients with cCTA (median 8.6 min, IQR 7.9–9.8 min) and those without (median 9 min with IQR 7.85–10.67 min) *p* *=* *0.10.* (B) *Door-to-needle time.* Addition of cCTA had no impact on the door-to-needle time for intravenous thrombolysis with cCTA (median 31 min, IQR 22–39 min) versus without cCTA (median 30 min, IQR 19–39 min), *p* *=* *0.37.* (C) *Door-to-groin puncture time.* Addition of cCTA had no impact on door-to-groin puncture time with cCTA (median 46.5 min, IQR 31.5–60 min) and without cCTA (median 47 min, IQR 27–59 min), *p* *=* *0.47.*

cCTA did not impact imaging or treatment time metrics (Figure 3). Total CT time, defined as the time from first to last image, was unchanged with cCTA (median 8.6 min, interquartile range (IQR) 7.9–9.8 min) versus without (median 9 min, IQR 7.85–10.67 min), *p* *=* *0.10*, Figure 3(a). Similarly, there was no difference in door-to-needle time with cCTA (median 31 min, IQR 22–39 min) versus without (median 30 min, IQR 19–39 min), *p* *=* *0.37*, Figure 3(b). Finally, door-to-groin puncture time remained similar with cCTA (median 46.5 min, IQR 31.5–60 min) versus without (median 47 min, IQR 27–59 min), *p* *=* *0.47*, Figure 3(c).

#### Recruitment feasibility

All patients that met inclusion and exclusion criteria consented and were recruited into the study.

#### Safety

Safety outcome of additional radiation exposure was estimated from the dose-length product (DLP). The average DLP for additional chest imaging was 363.9 mGy.cm (IQR 301–603 mGy.cm), with a corresponding radiation dose of 5.10 mSv (IQR 4.21–8.44 mSv). This extra radiation exposure (≈5 mSv) carries a ≈0.025% lifetime risk of fatal cancer.^20^

### Secondary outcomes

#### Rates of detecting high-risk cardiac sources of embolism

High-risk cardiac sources of embolism were identified in 12 of 89 (13.5%) patients with a final diagnosis of stroke (Table 3). These consisted of cardiac thrombi, specifically LAA thrombus in 11/89 (12.4%) and LV thrombus in 1/89 (1.1%).

**Table 3.** cCTA detection of cardiac sources of emboli and other pathologies.

	*n*/*N* (%)
Patients with high-risk cardiac source	12/89 (13.5%)
LA, LAA, or ventricular thrombus	12/89 (13.5%)
Infective endocarditis	0/89 (0%)
Atrial myxoma	0/89 (0%)
Foramen ovale with thrombus	0/89 (0%)
Additional incidental findings	
Pulmonary embolism	7/89(7.9%)
Malignancy (new diagnosis or significant progression)	6/89 (6.7%)
Long segment left subclavian thrombus	1/89(1.1%)

LA: left atrium; LAA: left atrium appendage.

Of patients with a finding of suspected LAA thrombus on cCTA, 4/11 underwent confirmatory TEE, while seven did not due to the finding of new/existing AF. This was due to the physician’s decision that confirmation would not change management. All tests confirmed original cCTA findings. One patient had a finding of LV thrombus, confirmed by TTE.

Of patients in whom cardiac sources of embolism were identified, 7/12 (59%) had imaging graded as good for motion artifact, while 5/12 (41%) were graded as moderate. Contrast opacification was good in 11/12 (92%) patients and moderate in 1/12 (8%).

#### Rates of detecting non-cardiac pathologies (pulmonary, vascular, and mediastinal)

Incidental findings were found on cCTA (Table 3), the most common of which was an asymptomatic PE. This was found in 7/89 (7.9%) patients with confirmed ischemic stroke. None of the patients with incidental PE had a concurrent diagnosis of cancer. Lifelong anticoagulation was recommended by subspecialty services for all patients with incidental unprovoked PE.

PE was not always necessarily related to stroke etiology. Of patients with PE, 4/7 were found to have a patent foramen ovale (PFO) on TTE, with the combination of the two being stroke etiology in 3/7 patients (the other patient had a concurrent left ventricle (LV) thrombus, which was thought to be the primary etiology).

Another important incidental finding was that of cancer. This was found in 6/89 (6.7%) of patients. Of these, 4/6 were new diagnoses (three lungs, one lymphoma), and 2 were extensive progression which led to a change in treatment goals.

#### Impact on TOAST classification of stroke

**Figure 4.**
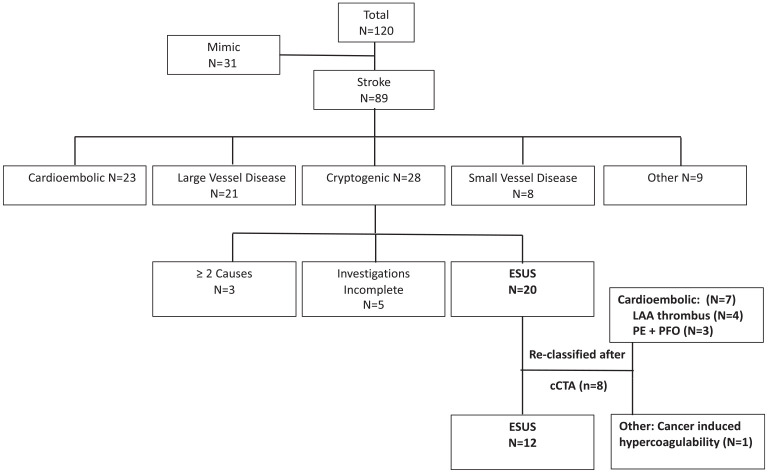
Etiological classification based on TOAST criteria after implementation of non–ECG-gated cCTA. Known stroke etiologies accounted for 61/89 (68.5%) stroke patients, with 28 (31.5%) patients retaining the diagnosis of cryptogenic stroke. Of these, 20 were ESUS. With our modified cCTA protocol, 8 ESUS patients had etiology reclassified, primarily into cardioembolic causes (LAA thrombus and PE with PFO.) Another patient’s stroke was attributed to cancer-induced thrombosis.

Figure 4 shows the breakdown of stroke etiology at discharge based on TOAST criteria.^21^

cCTA led to changes in the etiological classification of 8/89 stroke patients (9.0%) and reduced the number of ESUS patients from 20/89 (22.5%) to 12/89 (13.5%). Most reclassified patients were assigned to the cardioembolic group, with four showing LAA thrombus and three showing a combination of PE/PFO. The latter group may not have otherwise had a bubble study as part of their TTE, as we do not routinely perform this on patients over the age of 60 as they are not candidates for closure based on Canadian Guidelines.^22^ One patient had a new diagnosis of malignancy, and the etiology was attributed to cancer-associated thrombosis.

cCTA led to changes in management in 18/89 (20%) of stroke patients (see Supplemental Table 2). Most (12/18) had initiation of anticoagulation, while others had expedited work-up for new cancer (3/18) or transition to comfort-directed care for cancer progression (3/18).

## Discussion

In this single-center, prospective study featuring 120 patients presenting as Code Stroke, non–ECG-gated cCTA provided good to moderate quality images with regards to motion artifact and contrast opacification using a 64-slice CT scanner. This was achieved without significant delays in hyperacute stroke time metrics. It led to the identification of cardiac thrombi, as well as asymptomatic PE, and new/progressive malignancy. cCTA findings unmasked stroke etiology and contributed to changes in patient management.

### CCTA feasibility, safety, and utility

There has been recent emerging literature on cCTA as an alternative to TEE for the detection of cardiac thrombi.^14,15,23^ A recent meta-analysis on the detection of LAA thrombus by cCTA in patients with AF showed a high diagnostic accuracy of cCTA compared with TEE.^23^ Another meta-analysis assessing cCTA specifically in stroke patients showed high detection of cardiac thrombi, with pooled sensitivity and specificity of cCTA versus TEE of 86.0% (95% CI 65.6–95.2) and 97.4% (95% CI 95.0–98.7), respectively.^15^

In a recent study, ECG-gated cCTA in the setting of hyperacute stroke found a cardio-aortic source of embolism in 11.4% of patients on cCTA, compared with 4.9% on TTE.^12^ Specifically, cardiac thrombus was detected in 8% of patients.^24^ cCTA was performed as a separate study immediately after neuroimaging, with a median increased total scan time of 6 min.^12^ We chose non–ECG-gated cCTA, as it is seamlessly incorporated into current protocols and potentially prevents treatment delays.^15,25^ While we recognize this may not be comparable to TEE in diagnostic accuracy, we hypothesized that (1) diagnostic images could be possible, as cardiac sources can be detected on non-cardiac CT^26^ and (2) findings may improve the detection of embolic sources compared to current standards, as TEE is not routine in etiological workup as per most major guidelines.^9–11^ Our findings are supported by the ENCLOSE study,^19^ which reported a 12% yield of cardiac thrombi in patients with acute stroke or transient ischemic attack.

The additional radiation dosage of ≈5 mSv carries a ≈0.025% lifetime risk of fatal cancer, which is consistent with previous studies.^18^ With this risk profile, we believe non–ECG-gated cCTA is safe in the primarily elderly stroke population.

Overall, our study suggests easy implementation of modified imaging of the heart–brain axis, with adequate image quality in the majority of cases, without delay in diagnosis or treatment of hyperacute stroke. It provides a basis for larger studies at centers with similar technology and radiology expertise.

### CCTA for detection of pathologies and impact stroke classification and patient management

#### CCTA for detection of cardiac sources of embolism

Results demonstrate that high-risk cardiac sources of emboli can be visualized with non–ECG-gated cCTA in 13.5% of ischemic stroke patients. This is similar to other smaller studies on non–ECG-gated cCTA in stroke patients (detection rate ~15%).^17,25^

#### CCTA for identification of concomitant PE and acute ischemic stroke

cCTA led to the identification of PE in 7.9% of stroke patients. PEs detected were asymptomatic, despite moderate/high clot burden. Anticoagulation was recommended by subspecialists, and none of the patients had treatment complications. To our knowledge, this is the first and largest study to highlight concomitant PE and hyperacute ischemic stroke, as previous data on this is limited to case reports or very small case series for a total of 17 patients.^27–31^ While interesting, our data cannot be used to derive true incidence and prevalence rates due to its non-consecutive enrollment and small numbers. In a retrospective study from China, incidental PE was found in 1% of the stroke inpatient population,^32^ with most not mentioned in the initial radiology report. Almost all were high conspicuously and met the criteria for potential treatment. Incidental PE seems to be a common finding in CT examinations, with reports of 6.6% in the non-oncologic population.^33^ Overall, studies suggest PE is not rare in the setting of hyperacute stroke, which is not surprising given shared common risk factors of hypercoagulability, age, malignancy as well as causal pathways of paradoxical embolism.

#### CCTA impact on TOAST classification

Non–ECG-gated cCTA reduced at discharge ESUS population from 22.5% to 13.5%. Approximately 17% of ischemic strokes meet ESUS criteria (range 16–21%) based on registries.^34^ The assumption that these strokes were thromboembolic led to the hypothesis that direct oral anticoagulants (DOACs) would improve secondary prevention. However, subsequent clinical trials suggested that indiscriminate anticoagulation is neither effective nor safe.^3,4^ Attempts at parsing the heterogenous ESUS patients to refine a population that may benefit from anticoagulation are underway.^35^ Selective exclusion of ESUS patients with a low likelihood of developing AF has also been suggested by the AF-ESUS score.^36^ Advanced diagnostic techniques such as cancer screening and advanced vascular and cardiac imaging have also been proposed.^37^ In one study of 61 consecutive ESUS patients^38^, approximately half had aortogenic/cardiogenic sources that were missed in initial TTE, potentially affecting therapeutic strategy in 1 in 7 ESUS patients. cCTA, may be an adjunct investigation with the potential to provide etiological diagnosis at the time of inciting event, allowing for targeted secondary prevention early, when recurrence rates are highest.

#### Study limitations

This study has several limitations. First, non-consecutive enrollment does not allow for accurate estimations of the diagnostic yield of non–ECG-gated cCTA for identification of cardioembolic sources. There may be some selection bias, as we did not include after-hours population due to the limitations at our center. In addition, not all patients with cCTA-suggested thrombus underwent confirmatory testing due to known AF. Finally, patients with confirmed LAA thrombus did not undergo subsequent Holter monitoring, which may have later unmasked etiology.

#### Interpretation

This single-center prospective cohort study suggests that non–ECG-gated cCTA can be easily incorporated into Code Stroke imaging without delays in treatment and provide sufficient diagnostic quality imaging of the heart and chest structures. It seems to be a useful addition to the early diagnostic workup of stroke and may modify therapeutic decision-making for secondary prevention.

#### Generalizability

This is a single-center study which limits its generalizability and needs replication to validate outcomes. Its protocol, however, was pragmatic and seamlessly incorporated into current stroke imaging at this comprehensive stroke center, without additional injection of contrast, delay in imaging acquisition or stroke treatment, and a small additional dose of radiation. Images, provided by a third-generation 64-slice CT scanner, were a useful addition to the early diagnostic workup of stroke. Larger studies, reproducibility, and standardized imaging protocols are required to confirm findings and explore this further.

## Supplemental Material

sj-pdf-1-wso-10.1177_17474930231193335 – Supplemental material for Non–ECG-gated cardiac CT angiography in acute stroke is feasible and detects sources of embolism Click here for additional data file. Supplemental material, sj-pdf-1-wso-10.1177_17474930231193335 for Non–ECG-gated cardiac CT angiography in acute stroke is feasible and detects sources of embolism by Peter Lee, Gurmohan Dhillon, Marina Pourafkari, Dominique DaBreo, Zardasht Jaff, Ramana Appireddy, Albert Jin, Lysa Boissé Lomax, Bryce A Durafourt, John Gordon Boyd, Amir Reza Nasirzadeh, Donatella Tampieri and Shirin Jalini in International Journal of Stroke
