# Supplementary material for: Non–ECG-gated cardiac CT angiography in acute stroke is feasible and detects sources of embolism
Source: Int J Stroke. 2023 Aug 22;19(2):189–98. doi: 10.1177/17474930231193335 (PMC10811964; doi:10.1177/17474930231193335)
Supplement: sj-pdf-1-wso-10.1177_17474930231193335 – Supplemental material for Non–ECG-gated cardiac CT angiography in acute stroke is feasible and detects sources of embolism [file sj-pdf-1-wso-10.1177_17474930231193335.pdf]

Supplementary Material : Supplemental Tables 1-2

Supplemental Table 1: Cardiac CTA Imaging Protocol

**Protocol Parameter**	**Feature**
CT Scanner	General Electric Revolution HD
Patient Position	Supine, head first, arms along body
Detector Coverage	40 mm
Individual Detector Width	0.625 mm
Tube Voltage	120 kV
Tube Current	Auto mA, Smart mA Minimum 130, Maximum 625
Pitch	1.531:1
Slice thickness	0.625 mm
IV Access	20G or larger
Contrast for CT Angiogram	Omnipaque 350, total 75mL, 5cc/sec, Prewarmed (37°C; 99°F)

Supplemental Table 2: Impact of cCTA Findings on Patient Management

Patient	cCTA Finding	Change in Management
Patient 1	LAA Thrombus	Anticoagulation
Patient 2	LAA Thrombus	Anticoagulation
Patient 3	LAA Thrombus	Anticoagulation
Patient 4	LAA Thrombus	Anticoagulation
Patient 5	PE + PFO	Anticoagulation
Patient 6	PE + PFO	Anticoagulation
Patient 7	PE + PFO	Anticoagulation
Patent 8	PE + LV thrombus	Anticoagulation
Patient 9	PE	Anticoagulation
Patient 10	PE	Anticoagulation
Patient 11	PE	Anticoagulation
Patient 12	Lymphoma (New)	Early diagnosis/ management
Patient 13	Lung Cancer (New)	Early diagnosis/ management
Patient 14	Lung Cancer (New)	Early diagnosis/ management
Patient 15	Cancer Progression	Comfort-directed care
Patient 16	Cancer Progression	Comfort-directed care
Patient 17	Cancer Progression	Comfort-directed care
Patient 18	Long subclavian thrombus	Anticoagulation
Patient 19	Ventricular Perforation by Pacemaker Lead	Urgent Lead Revision
